# Supplementary material for: Novel DNM1L variants impair mitochondrial dynamics through divergent mechanisms
Source: Life Sci Alliance. 2022 Aug 1;5(12):e202101284. doi: 10.26508/lsa.202101284 (PMC9354038; doi:10.26508/lsa.202101284)
Supplement: Supplementary file 2 [file LSA-2021-01284_Supplemental_Data_1.docx]

**Supplementary information**

**Novel *DNM1L* variants impair mitochondrial dynamics through divergent mechanisms.**

Kelsey A. Nolden^1*^, John M. Egner^1*^, Jack J. Collier^2,3^, Oliver M. Russell^2^, Charlotte L. Alston^2,4^, Megan C. Harwig^1^, Michael E. Widlansky^5^, Souphatta Sasorith^6^, Inês A. Barbosa^7^, Andrew G. L. Douglas^8,9^, Julia Baptista^10,11^, Mark Walker^12^, Deirdre E. Donnelly^13^, Andrew A. Morris^14^, Hui Jeen Tan^15^, Manju A. Kurian^16^, Kathleen Gorman^17,18^, Santosh Mordekar^19^, Charu Deshpande^20^, Rajib Samanta^21^, Robert McFarland^2,4^, R. Blake Hill^1^, Robert W. Taylor^2,4^ and Monika Oláhová^2^

1. Expanded View Results, Materials and Methods

1.1 Clinical case reports

*Patient 1*

Patient 1 (P1) (c.1201G>A, p.Gly401Ser), a female, was the first child of non-consanguineous parents. The mother has Factor V Leiden-associated thrombophilia and had suffered nine spontaneous early miscarriages. Labour was induced at 37 weeks gestation following concerns regarding intrauterine growth restriction and birth weight was 2.24kg (9^th^ centile). In early infancy she was troubled by feeding difficulties, gastro-oesophageal reflux, faltering growth and had a febrile seizure at 5 months. Ophthalmology assessment at five months of age showed jerky smooth pursuit eye movements but was within normal limits. There was concern regarding her development on account of hypotonia and delayed motor milestones. On developmental assessment at the age of 8 months, she was able to hold her head up but was unable to roll over completely from back to front, and she could only sit with support. She was non-dysmorphic with a head circumference of 41.5 cm (2^nd^-9^th^ centile) and a weight of 6.4 kg at 8 months (2^nd^-9^th^ centile), with a length measured at 6 months of 62.2 cm (2^nd^-9^th^ centile). At the age of 10 months, she was admitted to hospital following a 3-month history of deterioration with reduced feeding, tiredness, rapid breathing, sweating and pallor. On admission she had sinus tachycardia and a lactate of 7.0 mmol/L (normal range 0.7-2.1 mmol/L). An echocardiogram revealed severe dilated cardiomyopathy with a dilated left ventricle and concentric left ventricular hypertrophy with a trabeculated appearance especially over the apex. The left ventricle showed severe impairment of systolic function (ejection fraction 36%) along with diastolic dysfunction (left ventricular end diastolic diameter 26 mm). Her condition and cardiac function deteriorated, and her treatment became palliative. She passed away shortly after, aged 10 months. P1 has an older maternal half-brother who has a history of non-verbal autism and febrile seizures. Immunology, microbiology and virology investigations did not identify any underlying cause for her cardiomyopathy. Urine organic acid analysis showed mild ketonuria and significant dicarboxylic aciduria consistent with a fasting ketosis and raised 3-methylglutaconic acid, consistent with 3-methylglutaconic aciduria type IV. Notably, these changes were only transiently evident during her acute illness. Bloodspot acylcarnitines showed an increase in C2-carnitine and C4-OH-carnitine consistent with a ketogenic response to metabolic stress. Brain magnetic resonance imaging (MRI) showed no definite features of leukodystrophy or basal ganglia signal abnormality, although there was equivocal pallidal high signal and mild thinning of the corpus callosum. 

*Patient 2*

Patient 2 (P2) (c.1088G>A, p.Gly363Asp) was the first child of non-consanguineous parents and there was no family history of note on either side. Antenatally, there were significant concerns about growth which prompted delivery by caesarean section at 34+4 weeks gestation. Her birth weight was 1660 g (2^nd^-9^th^ centile) and head circumference was 31 cm (25^th^ centile). No resuscitation was required but she spent 4 weeks in the Special Care Baby Unit. At 17 weeks of age (corrected age 11 weeks) her weight was 3.89 kg (0.4^th^ centile) her height and head circumference were also on the 0.4^th^ centile. At 21 weeks (corrected age 15 weeks) she developed infantile spasms and was admitted to hospital where she had a respiratory arrest. She showed a slow response to steroids and adrenocorticotropic hormone (ACTH). Developmentally, there were concerns regarding vision and development, as she was not smiling, or fixing and following. On examination at 24 weeks, she had microcephaly, arched eyebrows with upslanting palpebral fissures, and micrognathia. She had increased tone, with clenched hands and adducted thumbs. Her condition deteriorated and she died at the age of 13 months. Investigations showed raised lactate in cerebrospinal fluid (CSF), 5.5 mmol/L (normal range 1.2-2.1 mmol/L), and plasma 4.6 mmol/L (normal range 0.7-2.1 mmol/L) rising to 7.0mmol/L after 24 hours. Brain MRI showed hypoplasia of the corpus callosum and mild benign external hydrocephalus. Electroencephalogram (EEG) indicated hypsarrthythmia and echocardiogram revealed mild left ventricular hypertrophy.

*Patient 3*

Patient 3 (P3) (c.687_689dupATT, p.Leu230dup) was born to healthy non-consanguineous parents. She has a healthy brother and a healthy, non-identical, twin sister. P3 first presented at 6 years of age with chronic ataxia and mild learning difficulties. P3 developed left sided chorea and at 11 years of age, she had 3 generalised tonic-clonic convulsions. EEG showed frequent multifocal spike and wave activity with fronto-central emphasis and she was started on levetiracetam. Between 12 and 16 years of age, she had several admissions with status epilepticus or epilepsia partialis continua, with clonus of the left side of her face and arm. Between these episodes, she had moderately frequent focal seizures, such as left-sided facial clonic twitching without loss of consciousness. Various combinations of anticonvulsants were tried, along with a modified MCT-based ketogenic diet and subsequently vagus nerve stimulation. She developed nocturnal enuresis at 12 years of age, associated with an unstable bladder. Over the next 2 years, her gait deteriorated and she developed dysarthria. At 15 years, she required a keyboard or a scribe for writing. Over the next 2 years, she developed dysphagia requiring gastrostomy-feeding, a motor axonal neuropathy, pain in her right leg probably from increased tone with a fixed flexion contracture of her right knee. MRI showed myositis ossificans with oedema and pockets of fluid in the erector spinae, gluteal and thigh muscles. At 18 years of age, communication was limited, and she had dystonia and myoclonus. She died aged 20 years. She had mild microcephaly (head circumference between 0.4^th^ & 2^nd^ centiles). Cranial MRI was normal initially, but she subsequently showed cerebellar atrophy and mild atrophy of the cerebral hemispheres. There was persistent lymphopenia. Blood and CSF lactate concentrations were normal at 1.5 mmol/L (normal range 0.7-2.1 mmol/L) and 1.3 mmol/L (normal range 1.2-2.1 mmol/L), respectively, at 12 years of age, as were the glucose concentrations (5.6 and 4.2 mmol/L (normal range 4.0-7.0 mmol/L)). Urine organic acid and plasma amino acid analyses were also normal.

*Patient 4*

Patient 4 (P4) (c.2128A>G, p.Arg710Gly) was born to non-consanguineous parents and has a healthy older sibling. The mother has coeliac disease and there is no relevant family history. P4 was born at 35 weeks gestation by emergency caesarean section following a pregnancy that was complicated by gestational diabetes. He weighed 3.2 kg. He was hypoglycaemic and was admitted to the Neonatal Intensive Care Unit where a left-sided ptosis led to a diagnosis of a congenital Horner’s syndrome. He did not require artificial ventilation and bottle fed satisfactorily. He made good progress with motor skills initially, sitting at 8 months, but did not walk independently until 3-years when he had an ataxic gait. MRI of the brain at 1 and 2 years of age were normal. His left-sided ptosis was surgically corrected at 4 years of age at a time when “cryptogenic” epilepsy was diagnosed. Around this time, bilateral optic atrophy was identified; this remained stable with little impact on functional vision. MRI of the brain at the age of 5 years had subtle abnormalities in the parasagittal region of both hemispheres, suggestive of ulegyria. An extrapyramidal movement disorder began in his legs at 7 years and slowly progressed to his upper limbs by the age of 9 years. Scoliosis repair at age 8 years was followed by a loss of independent mobility. A severe aspiration pneumonia at 9 years of age required intensive care support and following videofluoroscopic confirmation of dysphagia he had a PEG inserted. Later that year electromyography and nerve conduction studies showed a severe underlying sensory-motor peripheral neuropathy, along with findings indicative of a superimposed demyelinating neuropathy. Regular (6 weekly) infusions of intravenous immunoglobulin were commenced with good effect and were continued for almost 4 years. Further spinal surgery at the age of 11 years was followed by marked functional deterioration and profound gut dysmotility to the point of intestinal failure requiring total parenteral nutrition, upon which he remained dependent, and latterly a requirement for opioid analgesia to manage abdominal pain. L-Dopa treatment had some positive impact on his dystonia, rigidity and involuntary movement. A clinical assessment at the age of 16 years revealed an occipitofrontal head circumference of 53.9 cm and a weight of 34.1 kg. Neurologically, he had no independent mobility and used an electric wheelchair. Both upper limbs were held in an adducted and flexed position at elbows and wrists. Fine motor skills were impaired, though he did manage to use phone and tablets. He had very restricted movements at knees and ankles bilaterally. Cognitively he remained good and communicated effectively. He had infrequent epileptic seizures with focal onset and secondary generalisation but did not take anticonvulsant medication. At the age of 17 years, he deteriorated over the course of several months with severe abdominal pain, increasingly frequent seizures and progressive encephalopathy. TPN was thought to be prolonging an intolerable life and, following discussion with the family and wider clinical team, was withdrawn 4 weeks prior to his death in a hospice.

*Patient 5*

Patient 5 (P5) (c.1201G>A, p.Gly401Ser) is the first child of non-consanguineous white European parents, who presented with early onset epileptic encephalopathy with two episodes of status epilepticus at the age of 2 years and 9 months. He was originally referred for global developmental delay with significant delay in motor skills, speech and to some extent intellectual skills. He presented with a prolonged convulsive seizure (status epilepticus), which was controlled with medication. He was intubated, ventilated, and transferred to ITU for further care. Detailed neurometabolic screening involving lactate and pyruvate in the cerebrospinal fluid and neuroimaging were all normal. He also presented with ongoing semirhythmic/variable intensity persistent twitching motion in his left upper limb. Initial hemiparetic posture involving both upper and lower limbs gradually improved and was resolved with only residual mild left upper limb monoparesis. He experienced regression of some of his developmental skills during his recovery and is now completely non-verbal. He was also quite lethargic and hypotonic with additional concerns regarding his swallowing that prompted referral for a gastrostomy. A year later, he had another episode of status epilepticus. Following recovery from two different episodes of status epilepticus, he was left with a semirepetitive dyskinesogenic high frequency movement disorder of variable intensity involving the upper limbs and face predominantly. He has a history of horizontal nystagmus. Repeat MRI imaging on this occasion showed disease progression with new bilateral high T2 and low T1 signal in the olivary nuclei of the medulla in keeping with cystic degeneration. There is less well defined T2 hyperintensity in the left thalamus. Neurometabolic screen involving lactate and pyruvate concentrations in the CSF, anti-neuronal antibodies and anti-NMDAR antibodies, plasma amino acids, urinary amino acids, organic acids, urine sialic acid screening was essentially normal.

1.2 Western blot analysis

Whole cell lysates from fibroblasts were lysed for 20 minutes on ice in lysis buffer [50 mM Tris-HCl pH 7.5, 130 mM NaCl, 2 mM MgCl_2_, 1 mM phenylmethanesulfonyl fluoride (PMSF), 1% Nonidet P-40 (v/v) and 1 × EDTA free protease inhibitor cocktail], denatured at 95°C for 5 minutes and separated by 12% SDS as previously described (Oláhová *et al.*, 2015). Following electrophoretic transfer of proteins to polyvinylidene difluoride (PVDF) membranes, immunoblotting was performed using antibodies against DRP1 (BD Transduction laboratories, 611113), Total OXPHOS Human WB Antibody Cocktail (Abcam, ab110411), SMCR7L (MID51) (ab89944), ß-actin (Cloud Clone Corp. CAB340Hu22) and GAPDH (Proteintech 60004-1-Ig).

For SDS-PAGE immunoblotting analysis, species appropriate horseradish peroxidase‐conjugated secondary antibodies (Dako) were used and all the primary antibodies were diluted 1:1000 except for ß-actin (1:10 000) and GAPDH (1:2000). Amersham ECL Prime Western Blotting Detection Reagent (GE Healthcare, Life Sciences) and The Bio‐Rad ChemiDoc MP with Image Lab software were used for protein visualisation and densitometry quantification of the blots.

1.3 BMH crosslinking

Control and patient (P4) primary fibroblasts were harvested at ~80% confluency, washed in 1x PBS and pellets were lysed in 200 μl lysis buffer [10 mM HEPES, 150 mM NaCl, 1 mM EGTA, 1% Triton-X (v/v), 1mM PMSF and 1 × EDTA free protease inhibitor cocktail] containing 1mM DMSO or 1mM bis‐maleimido‐hexane (BMH) cross-linker for 30 minutes at RT with gentle rotation. The reaction was inactivated by the addition of 20 mM DTT for 15 minutes at RT. Protein concentration of the supernatant following centrifugation of the lysates at 500 rcf for 5 minutes was determined by Bradford assay (BioRad) and 50 μg of each sample, denatured at 70°C for 10 minutes in the presence of NuPAGE™ LDS Sample Buffer (ThermoFisher), was loaded onto a NuPAGE 3-8% Tris Acetate Gel (Novex) and analysed by Western blotting.

**References**

Oláhová M, Hardy SA, Hall J, Yarham JW, Haack TB, Wilson WC, Alston CL, He L, Aznauryan E, Brown RM, et al (2015) *LRPPRC* mutations cause early-onset multisystem mitochondrial disease outside of the French-Canadian population. Brain 138: 3503–3519

==========

Nolden *et al.* | **27**
