# Supplementary material for: Novel DNM1L variants impair mitochondrial dynamics through divergent mechanisms
Source: Life Sci Alliance. 2022 Aug 1;5(12):e202101284. doi: 10.26508/lsa.202101284 (PMC9354038; doi:10.26508/lsa.202101284)
Supplement: Supplementary file 3 [file LSA-2021-01284_SdataFS2_FS5_FS7.pdf]

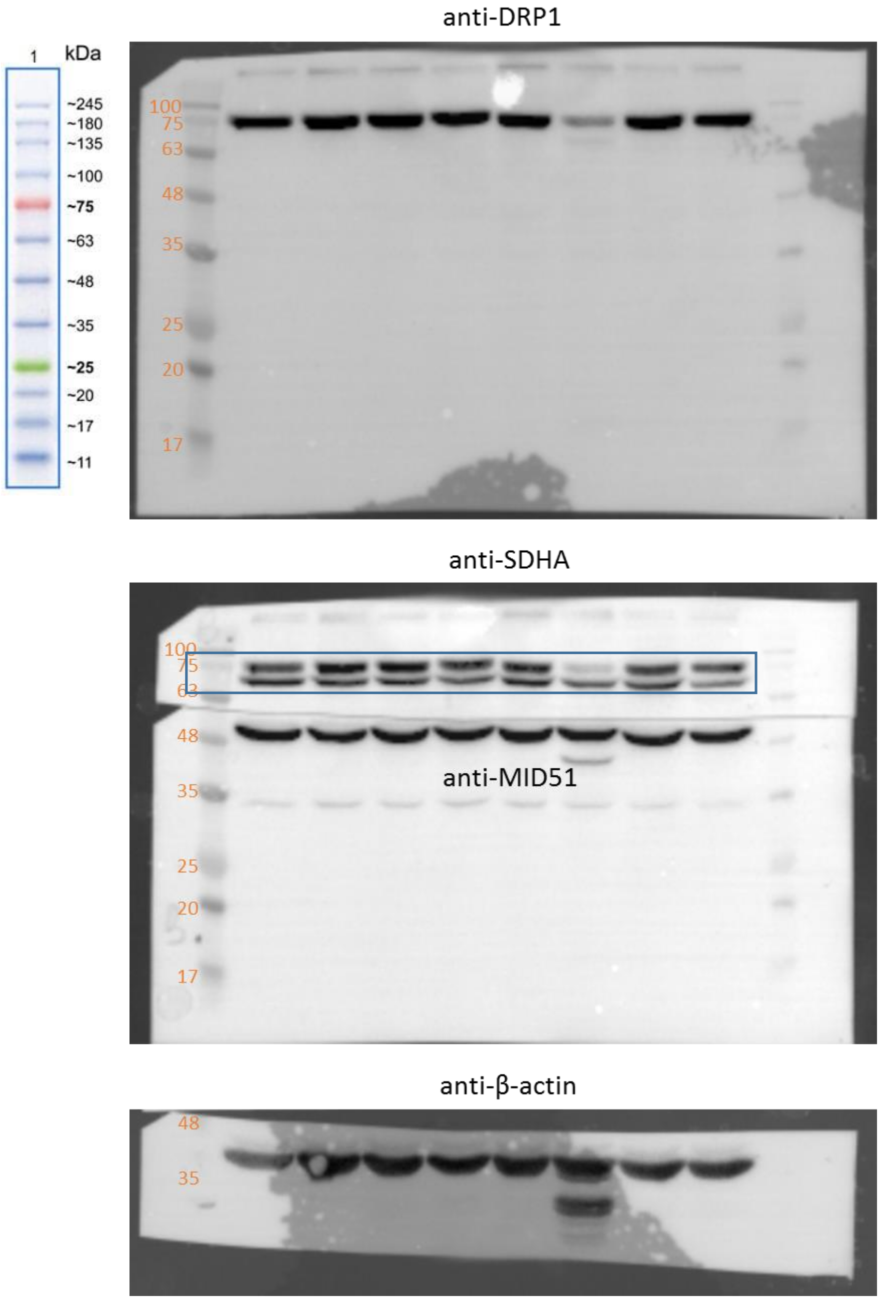

Figure S2. Steady-state levels of fission machinery proteins in DNM1L patient fibroblasts

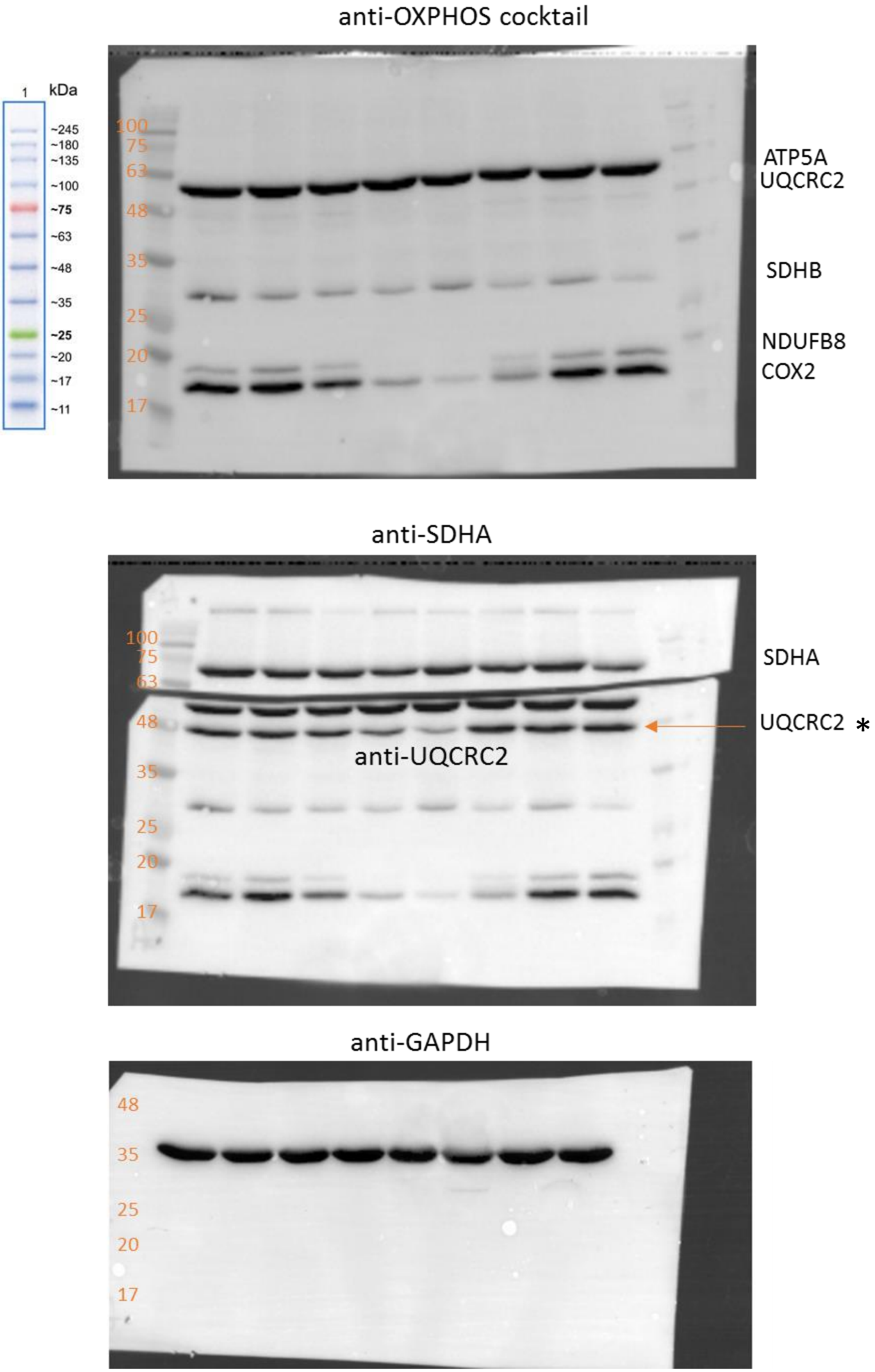

Figure S5. DNM1L patient fibroblasts demonstrate OXPHOS deficiencies.

*anti-UQCRC2 applied separately o/n 1:1000 dilution

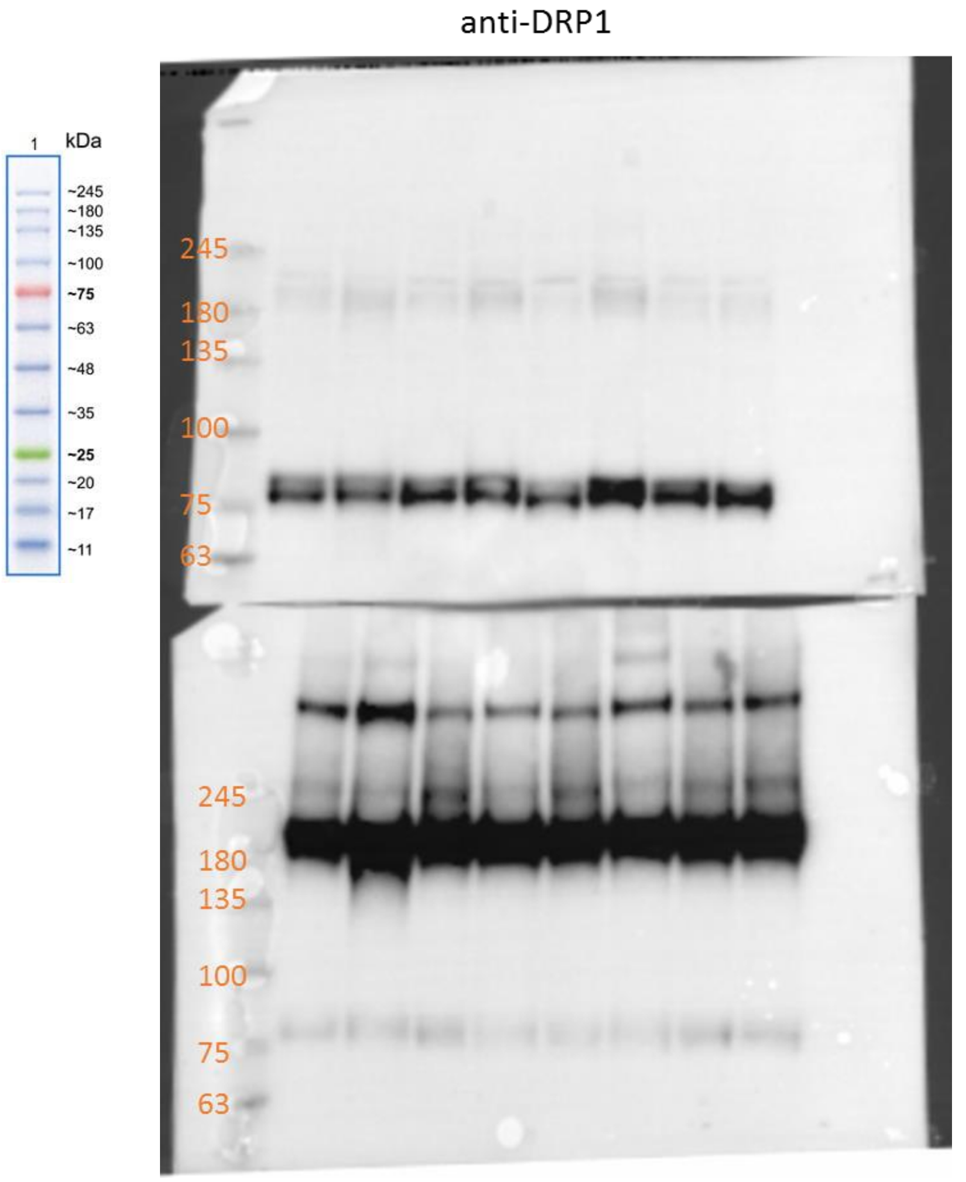

Figure S7. Analysis of DRP1 oligomers in DNM1L patient fibroblasts.

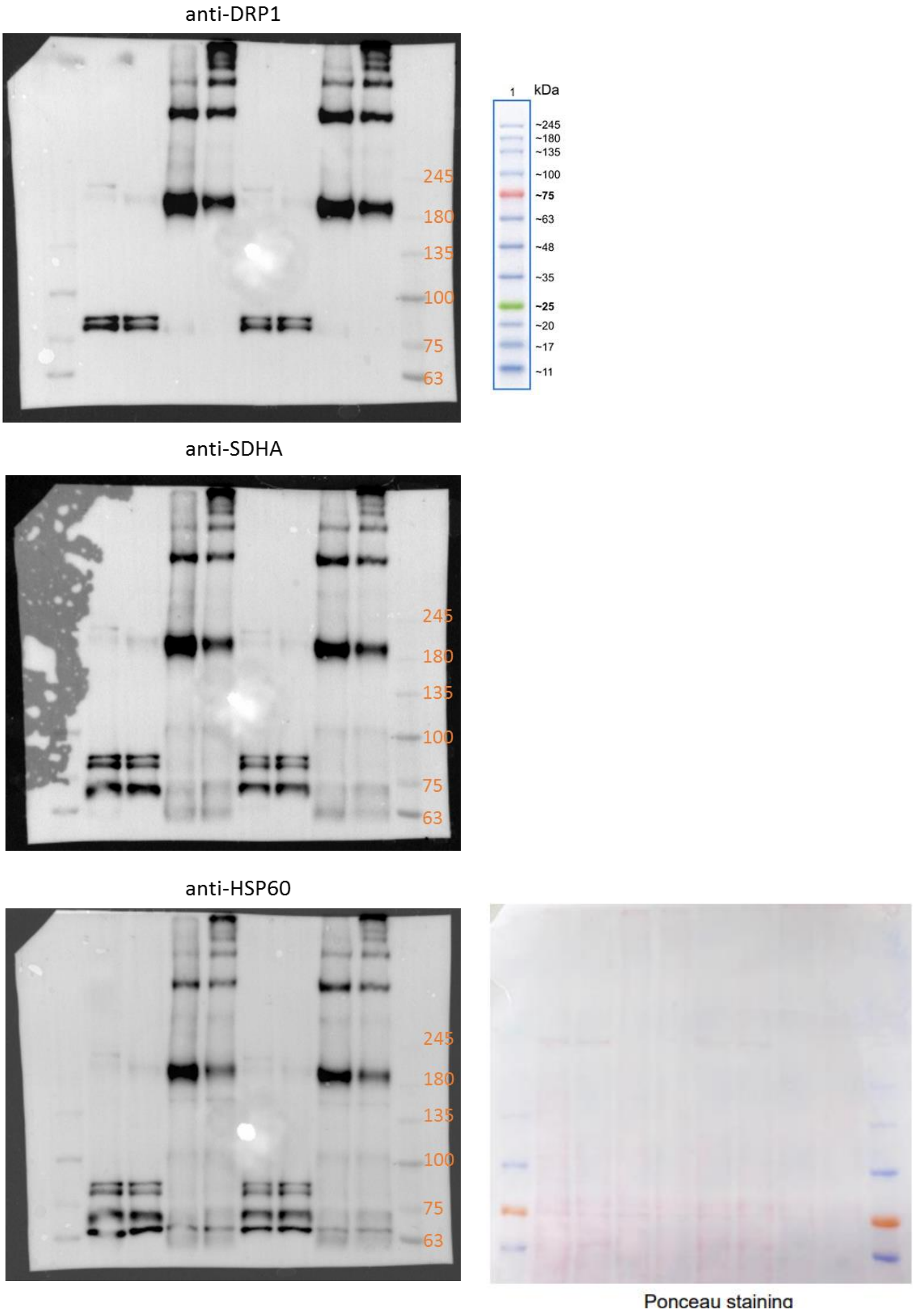

Figure S7. Analysis of DRP1 oligomers in DNM1L patient fibroblasts.
